# Supplementary material for: Glia instruct axon regeneration via a ternary modulation of neuronal calcium channels in Drosophila
Source: Nat Commun. 2023 Oct 14;14:6490. doi: 10.1038/s41467-023-42306-2 (PMC10576831; doi:10.1038/s41467-023-42306-2)
Supplement: Supplementary file 1 — Supplementary Information [file 41467_2023_42306_MOESM1_ESM.pdf]

Glia instruct axon regeneration via a ternary modulation of neuronal calcium channels in *Drosophila*

Supplementary Information

Supplementary Figures 1-8

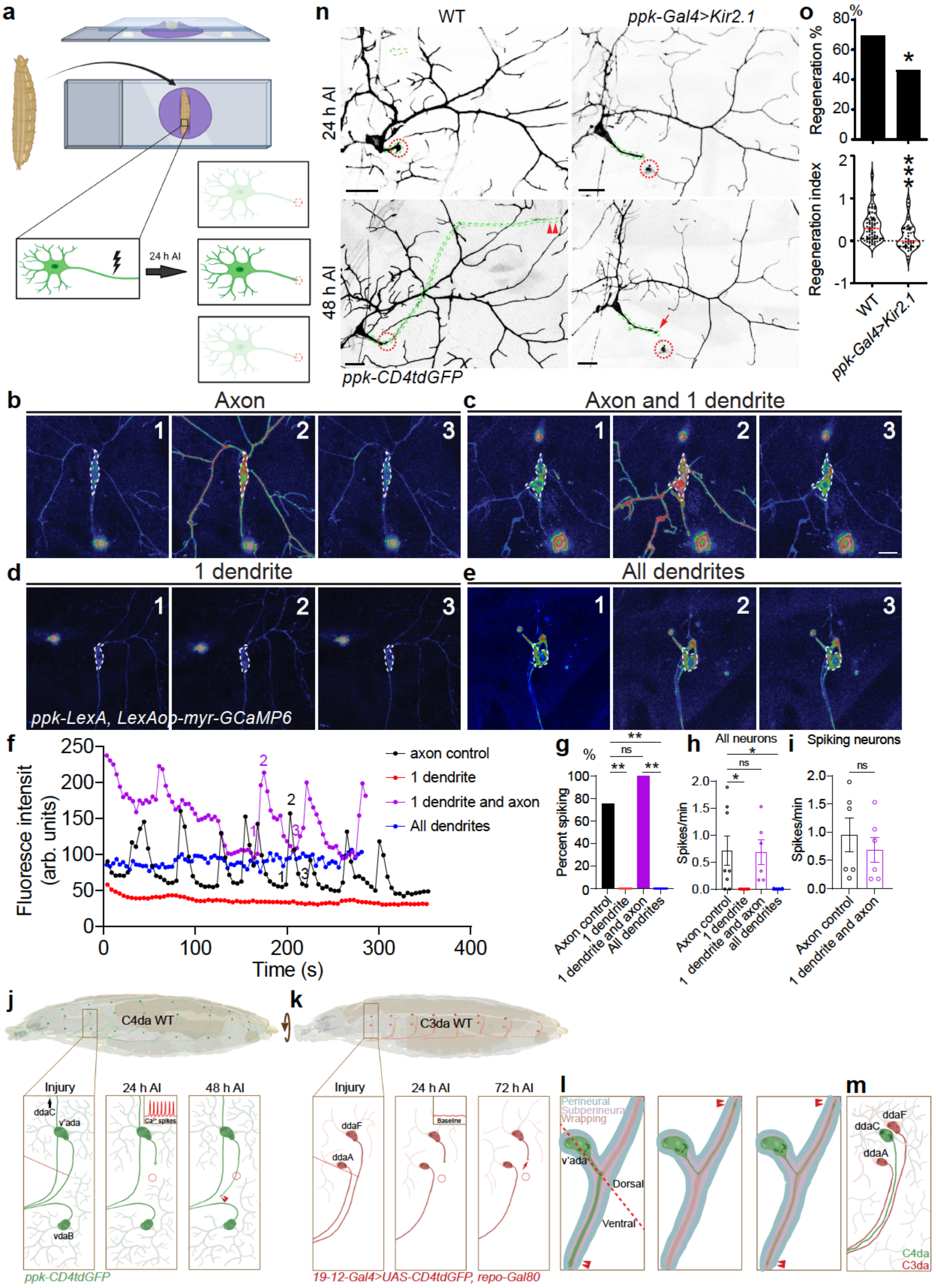

a
24 h AI
n
WT
ppk-Gal4>Kir2.1
24 h AI
48 h AI
ppk-CD4tdGFP
o
Regeneration %
Regeneration index
WT
ppk-Gal4>Kir2.1
b
Axon
c
Axon and 1 dendrite
d
1 dendrite
e
All dendrites
ppk-LexA, LexAop-myr-GCaMP6
f
Fluoresce intensit (arb. units)
Time (s)
axon control
1 dendrite
1 dendrite and axon
All dendrites
g
Percent spiking
h
All neurons
Spikes/min
i
Spiking neurons
Axon control
1 dendrite
1 dendrite and axon
All dendrites
j
C4da WT
Injury
24 h AI
48 h AI
ddaC
v'ada
vdaB
Ca²⁺ spikes
ppk-CD4tdGFP
k
C3da WT
Injury
24 h AI
72 h AI
ddaF
ddaA
Baseline
19-12-Gal4>UAS-CD4tdGFP, repo-Gal80
l
Perineural
Subperineural
Wrapping
v'ada
Dorsal
Ventral
m
ddaF
ddaC
ddaA
C4da
C3da

Supplementary Fig. 1 Ca^{2+} imaging paradigm, dendrite injury and axon regeneration quantification. (**a**) Ca^{2+} imaging set up for unanesthetized larvae. A larva is immobilized on a slide using clay. Axotomy is performed and Ca^{2+} imaging is performed at 24 h AI. The illustration in panel a was created using Biorender.com. (**b-e**) Dendrite injury does not induce Ca^{2+} transients. (**f**) Plots of the mean fluorescence intensity over time in the cell body. (**g**) Quantification of the percent of neurons showing Ca^{2+} spikes. N = 8, 6, 6, 6 neurons, P = 0.0097, 0.4725, 0.0097, 0.0022. (**h**, **i**) Quantification of the number of Ca^{2+} spikes per minute in all neurons (h, N = 8, 6, 6, 6 neurons, P = 0.0188, >0.9999, 0.0188) or spiking neurons (i, N = 6, 6 neurons, P = 0.5026). (**j-m**) Shown is a graphical depiction of *Drosophila melanogaster* larva accompanied by depictions of C4da and C3da, highlighting their consistent morphology. (**j**) C4da neurons, which reside along the body wall, are shown in green. The ddaC, v'ada and vdaB C4da neurons, are shown along with a brief description of the 2-photon injury timeline. The red dotted line is meant to depict the 2-photon laser injury and subsequent injury site. Notably, at 24 h AI, Ca^{2+} transients begin to occur; at 48 h AI, regrowth of injured axons has occurred. (**k**) C3da neurons, which also reside along the body wall, are shown in red. Similarly, the injury and injury site are depicted. C3da neurons, however, do not show the same Ca^{2+} transients at 24 h AI, and thus do not regrow in the span of 72 h AI. (**l**) Both C4da and C3da neurons are enclosed in 3 layers of glia, as depicted in different colors. Importantly, the perineural and subperineural glia encase both the cell body and the axon, while the wrapping glia only cover the axon. Specifically shown is the v'ada in order to illustrate the distinct glia tract morphology. As shown in the three panels, the v'ada may grow dorsally or ventrally to the cell body (upwardly or downwardly in this depiction) within the glia tracts. (**m**) Depicted is a bundle of neurons including both C4da and C3da neurons. The close proximity allows for direct comparisons between the two cell types, particularly with regard to gene

expression and axon regeneration. (**n**) C4da neuron overexpression of Kir2.1 reduces axon regeneration. The dashed circle marks the injury site. Arrowheads mark the regrowing axon tip and arrow marks the stalled axon. (**o**) Quantification of axon regeneration by regeneration percentage and regeneration index. $N = 72$, 43 neurons, $P = 0.0183$ (top), 0.0004 (bottom). $^*P < 0.05$, $^{**}P < 0.01$, $^{***}P < 0.001$, Fisher's exact test, two-sided (g and o top), unpaired Student's t-test, two-sided (i and o bottom), one-way ANOVA followed by Holm-Sidak's test (h). Scale bar = 20 μm. Source data are provided as a Source Data file.

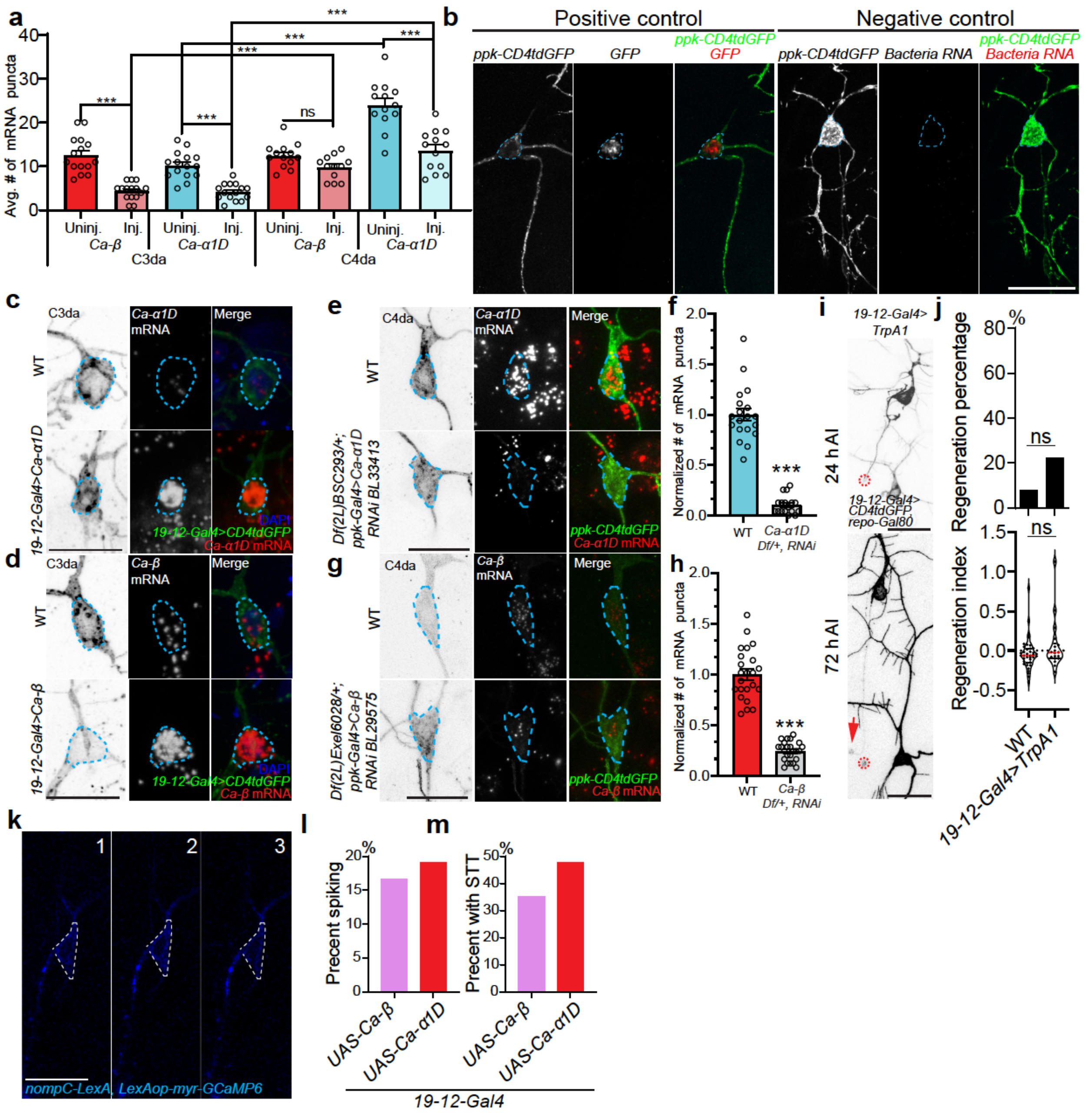

Supplementary Fig. 2 Controls for RNAscope and expression analyses of Ca-α1D and Ca-β.

(**a**) Quantification of the RNAscope analysis for Ca-α1D and Ca-β in C3da and C4da neurons with or without injury. *N* = 15, 15, 15, 15, 13, 13, 13, 13 neurons. (**b**) General positive and negative controls for RNAscope. A *GFP* probe specifically recognizes the *GFP* transcripts in the soma of C4da neurons expressing *ppk-CD4tdGFP*. A probe against *Bacteria RNA* does not show specific

labeling in fly larvae. (**c, d**) Positive controls for *Ca-α1D* and *Ca-β* mRNA using overexpression in C3da neurons and showing an increased number of puncta compared to WT. (**e-h**) Negative controls for *Ca-α1D* and *Ca-β* mRNA in C4da neurons. (e) A heterozygous deficiency covering Ca-α1D and C4da neuron targeted RNAi showed a decreased number of puncta. (f) Quantification of the number of *Ca-α1D* mRNA puncta, normalized to the WT average. $N = 19, 23$ neurons, $P = <0.0001$. (g) A heterozygous deficiency covering Ca-β and C4da neuron targeted RNAi showed a decreased number of puncta. (h) Quantification of the number of *Ca-β* mRNA puncta, normalized to the WT average. $N = 17, 24$ neurons, $P = <0.0001$. (**i**) Overexpression of TrpA1 in C3da neurons at the non-activating temperature did not increase regeneration beyond WT levels. An arrow marks the stalled axon tip and dashed circle marks the injury site. (**j**) Quantification of C3da axon regeneration with regeneration percentage and regeneration index with overexpression of TrpA1. $N = 38, 18$ neurons, $P = 0.1949$ (top), 0.2246 (bottom). (**k-m**) Overexpression of Ca-α1D or Ca-β alone slightly increases STT, but to a lesser extent than the co-overexpression. (k) An example of Ca-α1D overexpression is shown. (l) Quantification of the spiking percentage. (m) Quantification of the STT percentage. $N = 17$ and 21 neurons. $^*P < 0.05$, $^{**}P < 0.01$, $^{***}P < 0.001$, one-way ANOVA followed by Tukey's test (a), Fisher's exact test, two-sided (j top), two-tailed unpaired Student's t-test (f, h, j bottom). Scale bar = 20 μm. Source data are provided as a Source Data file.

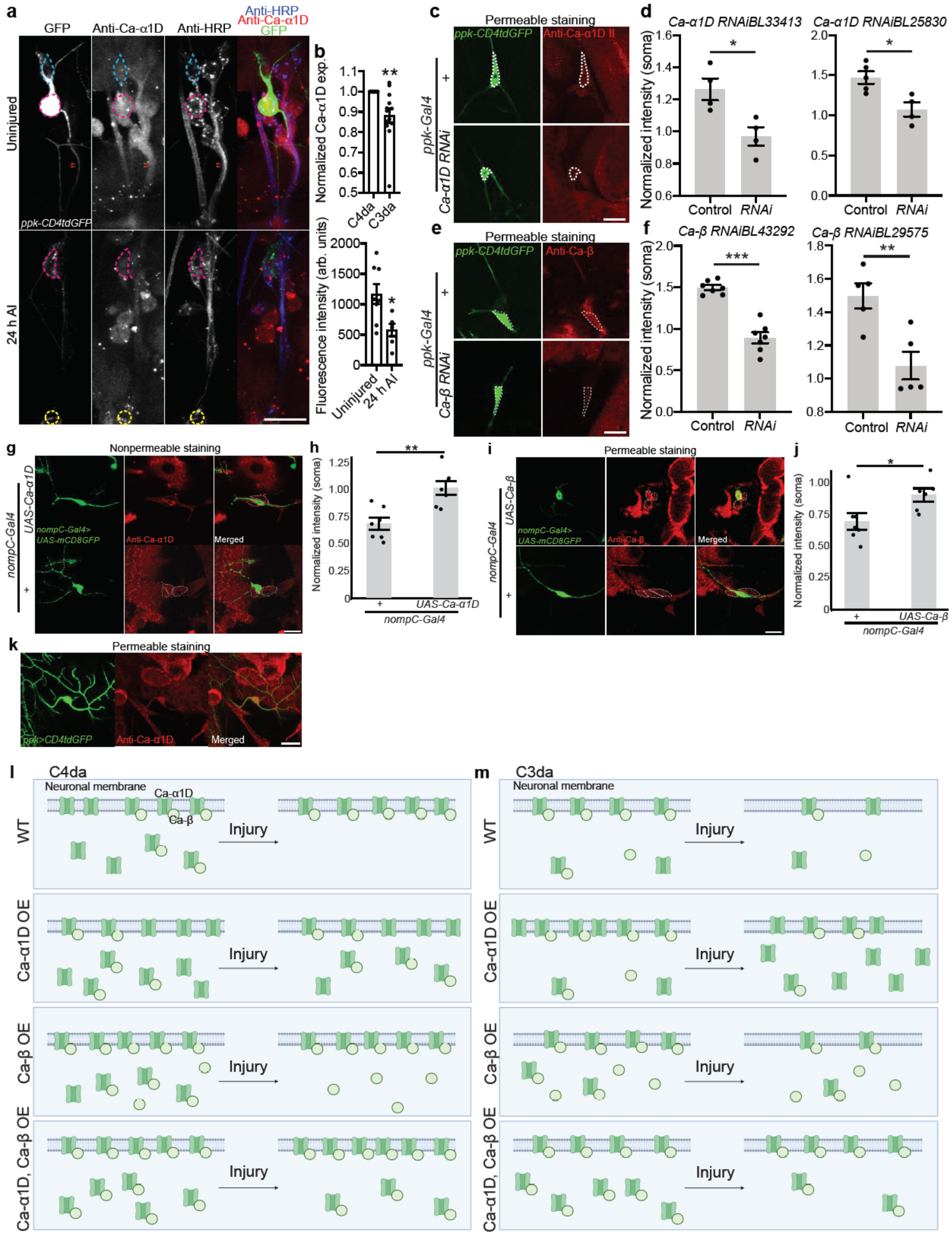

Supplementary Fig. 3 Control and validation of Ca-α1D and Ca-β antibodies. (a, b)

Expression of Ca-α1D by a published Ca-α1D antibody. (a) Ca-α1D protein is present in the soma (pink dashed circle), axon (arrowheads) and dendrites of C4da neurons. Ca-α1D is also present in C3da neurons (teal dashed circle). Axotomy reduces Ca-α1D expression in C4da neurons. The injury site is marked by the yellow dashed circle. (b) Top, quantification of Ca-α1D expression in the soma of C4da and C3da neurons. Mean fluorescence intensity is normalized to that of C4da neurons. $N = 13$ neurons, $P = 0.0024$. Bottom, Mean fluorescence intensity for Ca-α1D in C4da neurons with or without injury. $N = 8, 6$ neurons, $P = 0.0293$. (**c**, **d**) Verification of Ca-α1D expression with a second antibody and negative control in C4da. (**c**) Ca-α1D is present in C4da neurons. (**d**) Quantification of Ca-α1D level in the soma of control and C4da neuron-specific knockdown of Ca-α1D using two independent RNAis. $N = 4, 4, 5, 4$ neurons, $P = 0.0160$ (left), 0.0118 (right). (**e**, **f**) Verification of Ca-β expression with an antibody and negative control in C4da. (e) Ca-β is present in C4da neurons. (f) Quantification of Ca-β level in the soma of control and C4da neuron-specific knockdown of Ca-β using two independent RNAis. $N = 7, 7, 5, 5$ neurons, $P = <0.0001$ (left), 0.0057 (right). (**g, h**) Verification of Ca-α1D expression with a second antibody and positive control in C3da neurons. (g) Representative picture illustrates that Ca-α1D level on the soma plasma membrane of control and Ca-α1D-overexpressing C3da neurons, in the nonpermeable staining condition. (h) Quantification of Ca-α1D level on the soma plasma membrane of control and Ca-α1D-overexpressing C3da neurons in the permeable staining condition. N = 7, 7 neurons, $P = 0.0020$. (**i, j**) Verification of Ca-β expression with an antibody and positive control in C3da neurons. (i) Representative picture illustrates that Ca-β level in the soma of control and Ca-β-overexpressing C3da neurons. (j) Quantification of Ca-β level in the soma of control and Ca-β-overexpressing C3da neurons. N = 7, 6 neurons, $P = 0.0351$. (**k**)

Representative image of Ca-α1D expression using antibody in a permeabilized cell. (**l, m**) Graphical representation of the Ca-β/Ca-α1D ratio hypothesis. The illustration in panels l and m was created using Biorender.com. *P < 0.05, **P < 0.01, ***P < 0.001, Wilcoxon test (b top), Mann-Whitney test (b bottom), two-tailed unpaired Student's t-test (d, f, h, j). Scale bar = 20 μm. Source data are provided as a Source Data file.

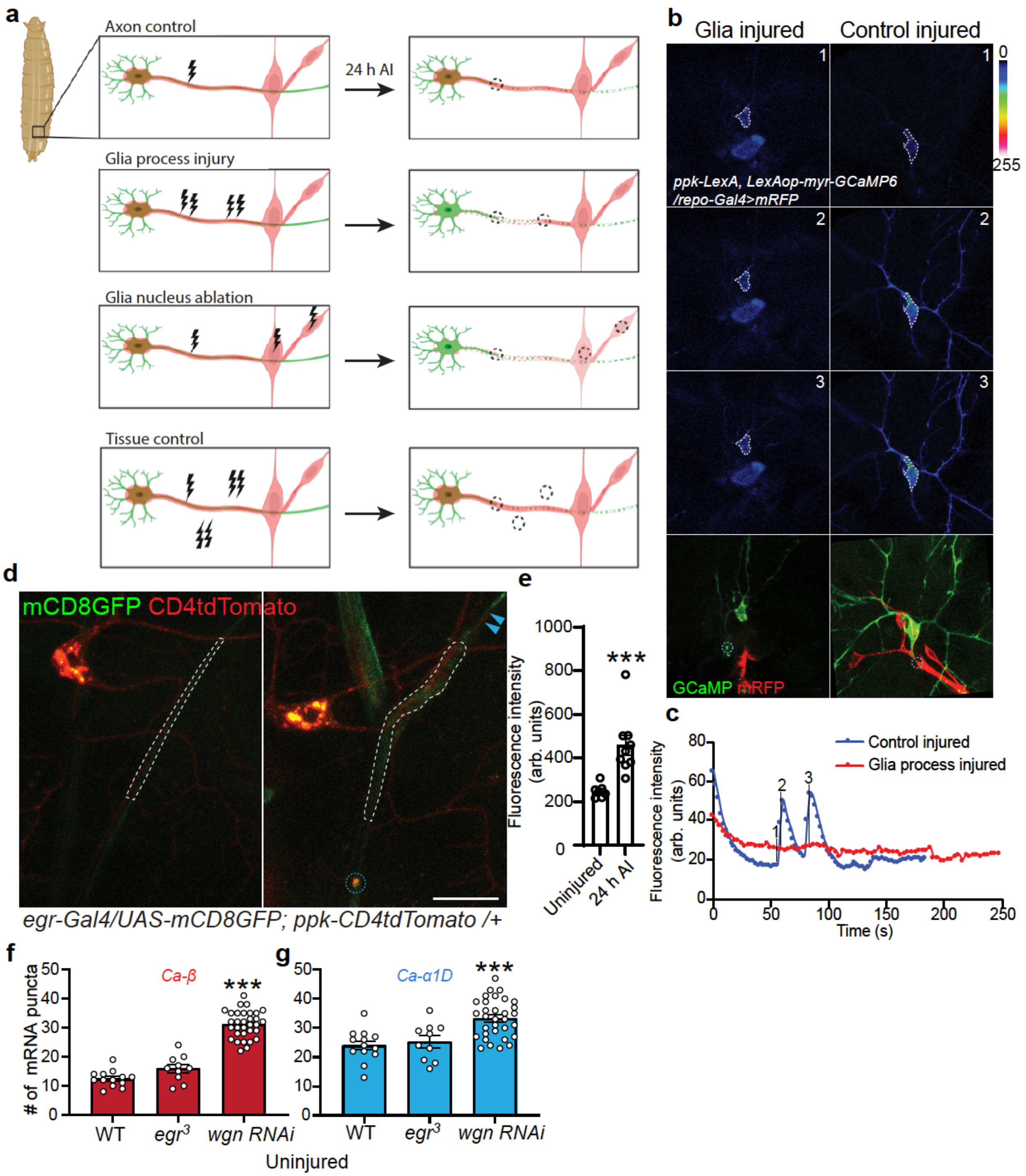

Supplementary Fig. 4 Glia injury and egr-Gal4 expression. (**a**) The glia injury paradigm. The illustration in panel a was created using Biorender.com. (**b**, **c**) Glia injury reduces axotomy-induced Ca^{2+} transients. (b) Compared to axon only injury in which glia still wrap the C4da neuron soma and axon, injury to the glial processes leads to unwrapping of the soma and axon, and results

in reduced Ca^{2+} transients. (**c**) Plot of the mean intensity of the soma. (**d**, **e**) *egr* expression is assessed by egr-Gal4 driving a mCD8GFP reporter. (d) The injury site is demarcated by the dashed circle. Arrowheads show the regrowing axon tip. (e) Quantification of GFP fluorescence intensity in the area outlined by the white dashed line in d. $N = 7, 9$ neurons, $P = 0.0003$. (**f, g**) Quantification of *Ca-α1D* and *Ca-β* mRNAs in uninjured C4da neurons after *egr* or *wgn* LoF. $N = 13, 10, 32, 13, 10, 32$ neurons, $P = <0.0001, <0.0001$. ***$P < 0.001$, one-way ANOVA followed by Tukey's test, (f, g) Mann Whitney test (e). Scale bar = 20 μm. Source data are provided as a Source Data file.

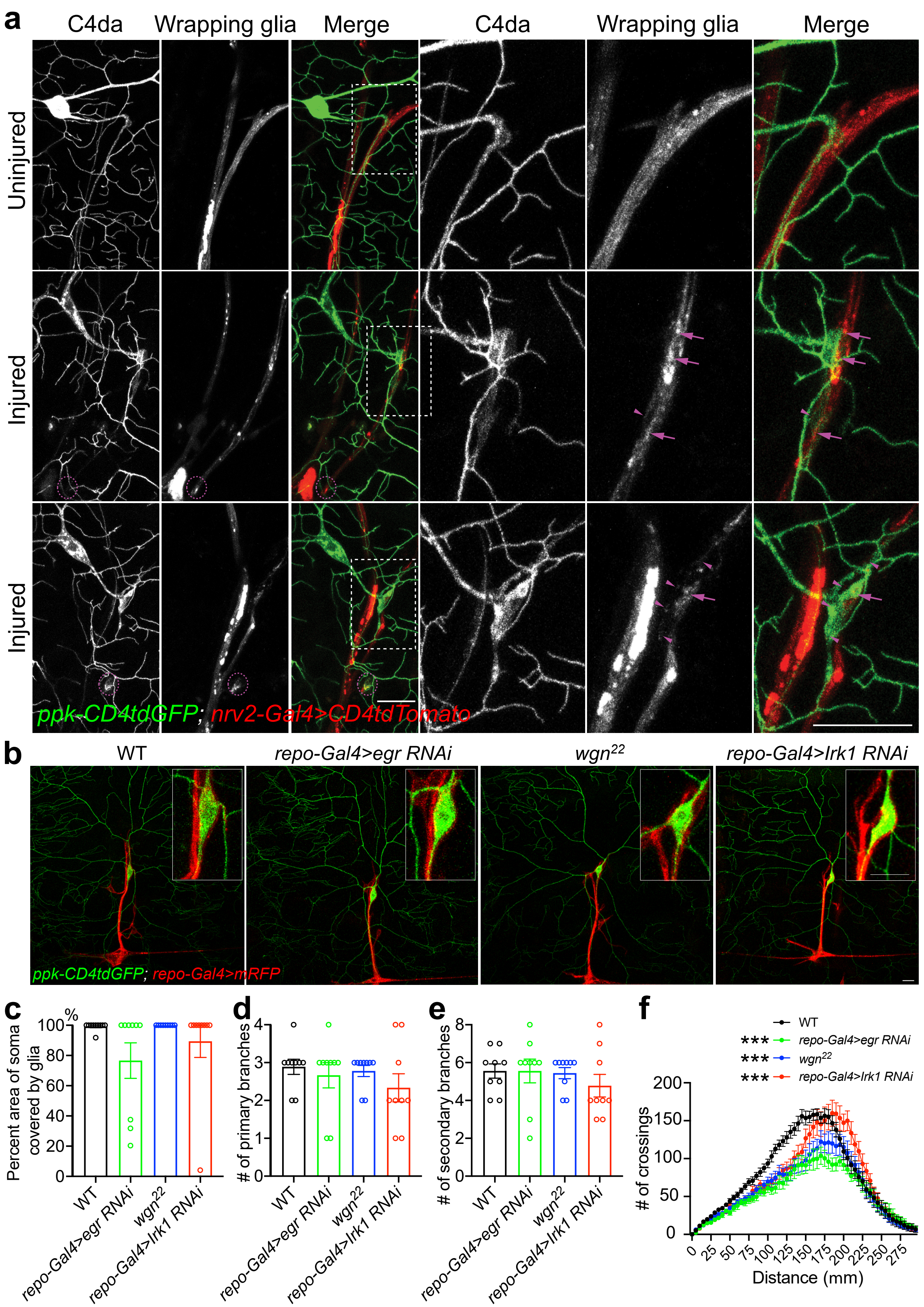
a
C4da
Wrapping glia
Merge
C4da
Wrapping glia
Merge
Uninjured
Injured
Injured
ppk-CD4tdGFP; nrv2-Gal4>CD4tdTomato
b
WT
repo-Gal4>egr RNAi
wgn22
repo-Gal4>Irk1 RNAi
ppk-CD4tdGFP; repo-Gal4>mRFP
c
Percent area of soma covered by glia
%
100
80
60
40
20
0
WT
repo-Gal4>egr RNAi
wgn22
repo-Gal4>Irk1 RNAi
d
# of primary branches
4
3
2
1
0
WT
repo-Gal4>egr RNAi
wgn22
repo-Gal4>Irk1 RNAi
e
# of secondary branches
8
6
4
2
0
WT
repo-Gal4>egr RNAi
wgn22
repo-Gal4>Irk1 RNAi
f
WT
*** repo-Gal4>egr RNAi
*** wgn22
*** repo-Gal4>Irk1 RNAi
# of crossings
200
150
100
50
0
0
25
50
75
100
125
150
175
200
225
250
275
Distance (mm)

Supplementary Fig. 5 Glial remodeling after axotomy, dendrite morphology after LoF of egr-wgn or Irk1. (**a**) Glial processes around the regenerating axon appear to undergo remodeling. Wrapping glia change from their even distribution surrounding uninjured axons to a clustered appearance (arrows) after axotomy and occasionally extend thin processes that contact the axon tip (arrowheads). The injury site is demarcated by the dashed circle. (**b**) Representative images of C4da neurons and glia from the various genotypes. The soma region is further shown in the white box. (**c-f**) Quantifications of glia wrapping, number of primary and secondary dendritic branches, and dendrite complexity with the sholl analysis. N = 10, 9, 9, 9 neurons (c), 9 neurons (d and e), 19, 9, 12, 9 neurons (f). ***P < 0.001, one-way ANOVA followed by Dunn's test (c-e), two-way ANOVA followed by Tukey's test (f). Scale bar = 20 µm. Source data are provided as a Source Data file.

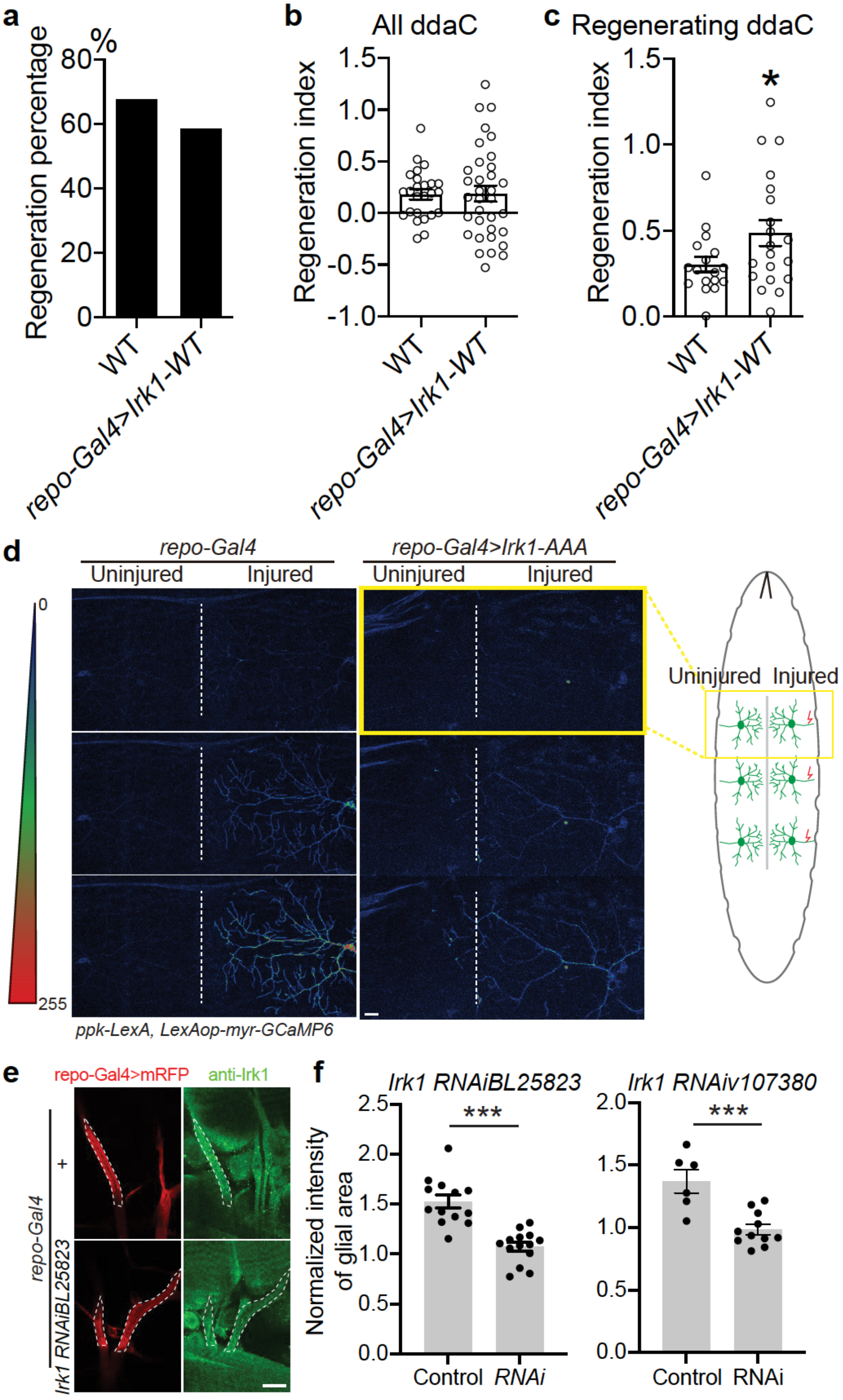

a
Regeneration percentage
%
80
60
40
20
0
WT
repo-Gal4>Irk1-WT
b
All ddaC
Regeneration index
1.5
1.0
0.5
0.0
-0.5
-1.0
WT
repo-Gal4>Irk1-WT
c
Regenerating ddaC
Regeneration index
1.5
1.0
0.5
0.0
*
WT
repo-Gal4>Irk1-WT
d
repo-Gal4
repo-Gal4>Irk1-AAA
Uninjured
Injured
Uninjured
Injured
0
255
Uninjured
Injured
ppk-LexA, LexAop-myr-GCaMP6
e
repo-Gal4>mRFP
anti-Irk1
repo-Gal4
+
Irk1 RNAiBL25823
f
Irk1 RNAiBL25823
Irk1 RNAiv107380
Normalized intensity of glial area
2.5
2.0
1.5
1.0
0.5
0.0
***
Control
RNAi
2.0
1.5
1.0
0.5
0.0
***
Control
RNAi

Supplementary Fig. 6 Glial Irk1 overexpression enhances C4da neuron axon regeneration, while inhibiting glial Irk1 reduces axotomy-induced Ca^{2+} transients in C4da neurons, and Irk1 expression analysis by an Irk1 antibody. (**a-c**) Glial overexpression of Irk1-WT increases axon regrowth among the regenerating C4da neuron ddaC. *N* = 25, 34 neurons, *P* = 0.5886, 0.9383, (a, b) and 17, 20 neurons, *P* = 0.0452 (c). (**d**) C4da neurons show Ca^{2+} spikes after axonal injury in WT (right side), compared to uninjured side in the same larvae (left side). LoF of Irk1 by glial overexpression of Irk1-AAA reduces Ca^{2+} spikes. (**e**, **f**) Verification of Irk1 expression with an antibody. (e) Irk1 is present in glial cells. (f) Quantification Irk1 level in glial processes (dashed line) of control and glia-specific knockdown of Irk1 using two independent RNAis. *N* = 13, 14, 6, 11 fields of view, *P* = <0.0001 (left), 0.0005 (right). $^{*}P < 0.05$, $^{***}P < 0.001$, Fisher's exact test, two-sided (a), two-tailed unpaired Student's t-test (b, c, and f). Scale bar = 20 µm. Source data are provided as a Source Data file.

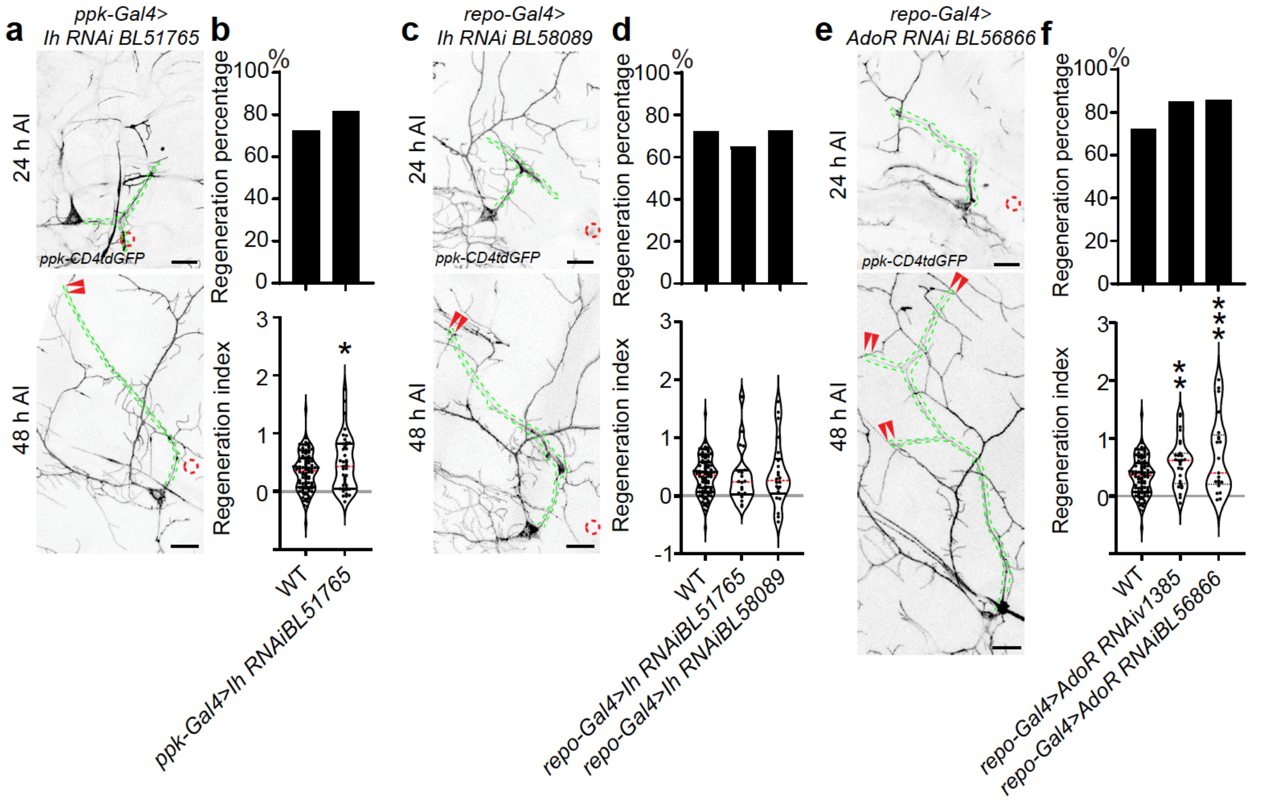

Supplementary Fig. 7 Neuronal versus glial knockdown of Ih or AdoR. (**a**) C4da neuron specific knockdown of *Ih* using a second RNAi (*ppk-Gal4Ih RNAi BL51765*) modestly increases axon regeneration. Arrowheads mark the regrowing axon tip and dashed circle marks the injury site. (**b**) Quantification of C4da axon regeneration with regeneration percentage and regeneration index with C4da neuron knockdown of *Ih*. $N = 94, 41$ neurons. (**c**) Glia-specific knockdown of *Ih* with two independent RNAis does not alter axon regeneration. (**d**) Quantification of C4da axon regeneration with regeneration percentage and regeneration index, with glia knockdown of *Ih*. $N = 94, 20, 35$ neurons. (**e**) Glia-specific knockdown of *AdoR* with two independent RNAis significantly increases axon regeneration. (**f**) Quantification of C4da axon regeneration with regeneration percentage and regeneration index, with glia knockdown of *AdoR*. $N = 94, 27, 21$ neurons. $*P < 0.05$, $**P < 0.01$, $***P < 0.001$, Fisher's exact test, two-sided (b top, d top and f

top), two-tailed unpaired Student's t-test (b bottom), one-way ANOVA followed by Holm-Sidak's test (d bottom and f bottom). Scale bar = 20 μm. Source data are provided as a Source Data file.

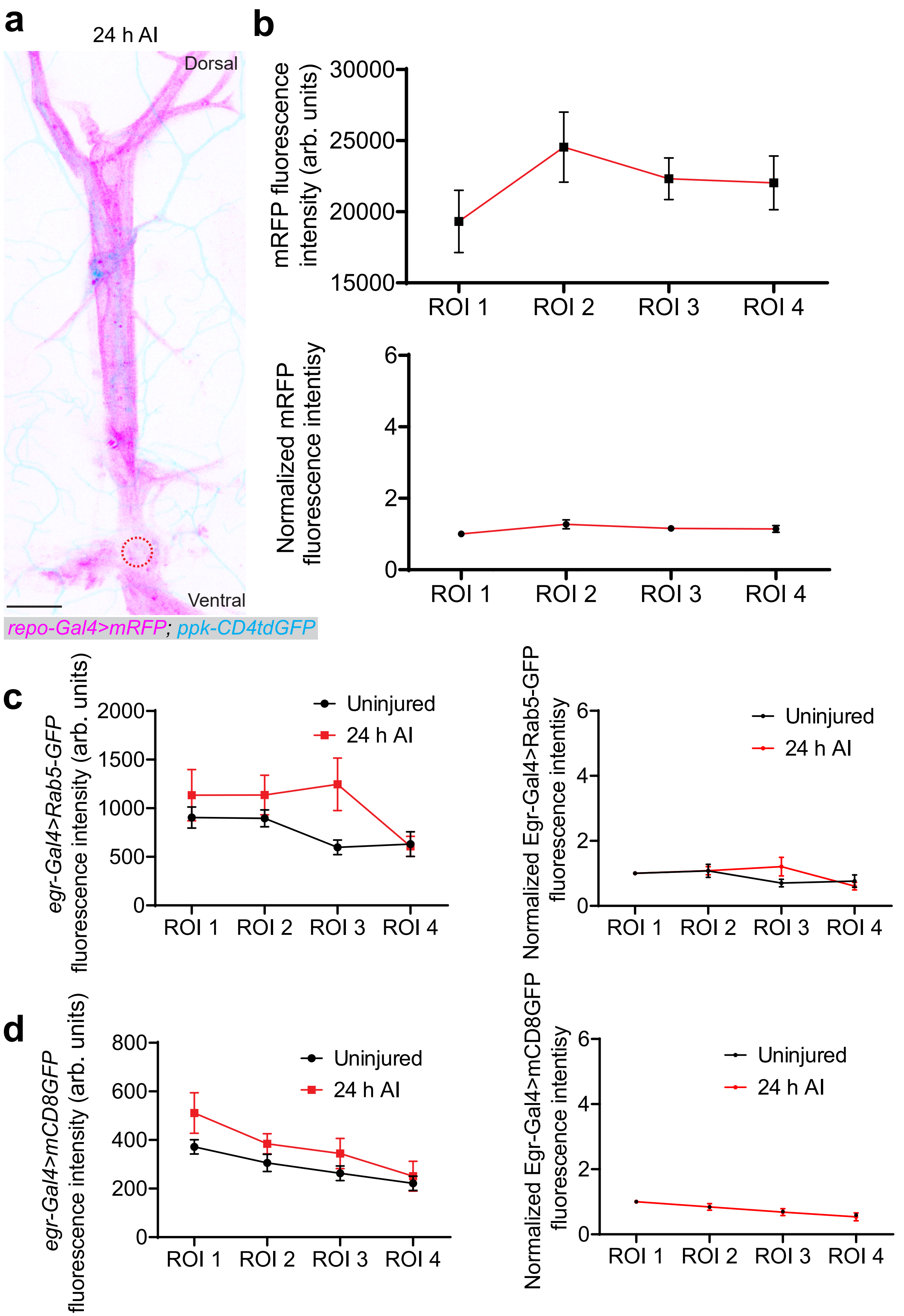

a
24 h AI
Dorsal
Ventral
repo-Gal4>mRFP; ppk-CD4tdGFP
b
mRFP fluorescence intensity (arb. units)
30000
25000
20000
15000
ROI 1
ROI 2
ROI 3
ROI 4
Normalized mRFP fluorescence intentisy
6
4
2
0
c
egr-Gal4>Rab5-GFP fluorescence intensity (arb. units)
2000
1500
1000
500
0
Uninjured
24 h AI
Normalized Egr-Gal4>Rab5-GFP fluorescence intentisy
d
egr-Gal4>mCD8GFP fluorescence intensity (arb. units)
800
600
400
200
Normalized Egr-Gal4>mCD8GFP fluorescence intentisy

Supplementary Fig. 8 mRFP and egr do not show a concentration gradient along C4da neuron axons. (**a**) Control for fluorescence gradient of glia using repo-Gal4 driving a mRFP reporter, showing no gradient in either direction. (**b**) Plots of fluorescence intensity over the four ROIs, with the bottom plot being normalized to the fluorescence of ROI 1. *N* = 16 neurons per ROI. (**c**, **d**) Plots of fluorescence intensity over the four ROIs by egr-Gal4 driving a Rab5-GFP reporter and an mCD8GFP reporter respectively are shown. The right-side plots are normalized to the fluorescence of the first ROI. No clear gradient can be seen. *N* = 7 (uninjured) and 7 (24 h AI) neurons per ROI (a), 7 (uninjured) and 9 (24 h AI) neurons per ROI (b). Source data are provided as a Source Data file.
