# Supplementary material for: Glia instruct axon regeneration via a ternary modulation of neuronal calcium channels in Drosophila
Source: Nat Commun. 2023 Oct 14;14:6490. doi: 10.1038/s41467-023-42306-2 (PMC10576831; doi:10.1038/s41467-023-42306-2)
Supplement: Supplementary file 2 — Reporting Summary [file 41467_2023_42306_MOESM2_ESM.pdf]

nature portfolio

Corresponding author(s): Yuanquan Song

Last updated by author(s): Sep 6, 2023

Reporting Summary

Nature Portfolio wishes to improve the reproducibility of the work that we publish. This form provides structure for consistency and transparency in reporting. For further information on Nature Portfolio policies, see our Editorial Policies and the Editorial Policy Checklist.

Statistics

For all statistical analyses, confirm that the following items are present in the figure legend, table legend, main text, or Methods section.

n/a	Confirmed	
☐	☒	The exact sample size (n) for each experimental group/condition, given as a discrete number and unit of measurement
☐	☒	A statement on whether measurements were taken from distinct samples or whether the same sample was measured repeatedly
☐	☒	The statistical test(s) used AND whether they are one- or two-sided<br>*Only common tests should be described solely by name; describe more complex techniques in the Methods section.*
☐	☒	A description of all covariates tested
☐	☒	A description of any assumptions or corrections, such as tests of normality and adjustment for multiple comparisons
☐	☒	A full description of the statistical parameters including central tendency (e.g. means) or other basic estimates (e.g. regression coefficient) AND variation (e.g. standard deviation) or associated estimates of uncertainty (e.g. confidence intervals)
☐	☒	For null hypothesis testing, the test statistic (e.g. F, t, r) with confidence intervals, effect sizes, degrees of freedom and P value noted<br>*Give P values as exact values whenever suitable.*
☒	☐	For Bayesian analysis, information on the choice of priors and Markov chain Monte Carlo settings
☒	☐	For hierarchical and complex designs, identification of the appropriate level for tests and full reporting of outcomes
☒	☐	Estimates of effect sizes (e.g. Cohen's d, Pearson's r), indicating how they were calculated

Our web collection on statistics for biologists contains articles on many of the points above.

Software and code

Policy information about availability of computer code

Data collection	Image data collection: Leica LAS X, Zeiss ZEN 2012
Data analysis	Image data analysis: ImageJ 1.52q, Statistical analyses: GraphPad Prism 8.

For manuscripts utilizing custom algorithms or software that are central to the research but not yet described in published literature, software must be made available to editors and reviewers. We strongly encourage code deposition in a community repository (e.g. GitHub). See the Nature Portfolio guidelines for submitting code & software for further information.

Data

Policy information about availability of data

All manuscripts must include a data availability statement. This statement should provide the following information, where applicable:

- Accession codes, unique identifiers, or web links for publicly available datasets
- A description of any restrictions on data availability
- For clinical datasets or third party data, please ensure that the statement adheres to our policy

Data supporting the findings of this study are provided within the paper and its supplementary information. Source data are provided with this paper.

Field-specific reporting

Life sciences study design

All studies must disclose on these points even when the disclosure is negative.

Sample size	The sample size and statistical tests were chosen based on previous studies with similar methodologies and the data met the assumptions for each statistical test performed (Song et al., Genes Dev 2012; Song et al., Nat Neurosci 2015; Song et al., Neuron 2019; Wang et al., Elife 2020; Li et al., Cell Metab 2020; Li et al., Nat Commun 2021). No statistical method was used in deciding sample sizes.
Data exclusions	No data were excluded from the analyses.
Replication	Each experiment was successfully reproduced at least three times and was performed on multiple days.
Randomization	Within genotypes, staged animals were allocated randomly. Samples were treated in a randomized manner during the analysis.
Blinding	Blinding was not applicable because the investigator who set up the experiments was the same person doing sample collection and analysis. However, each experiment was associated with proper controls, and samples were collected and analyzed under the identical conditions. Investigators performed phenotypic analyses without taking into account the genotype of sample groups.

Reporting for specific materials, systems and methods

We require information from authors about some types of materials, experimental systems and methods used in many studies. Here, indicate whether each material, system or method listed is relevant to your study. If you are not sure if a list item applies to your research, read the appropriate section before selecting a response.

Materials & experimental systems

n/a	Involved in the study	
☐	☒	Antibodies
☒	☐	Eukaryotic cell lines
☒	☐	Palaeontology and archaeology
☐	☒	Animals and other organisms
☒	☐	Human research participants
☒	☐	Clinical data
☒	☐	Dual use research of concern

Methods

n/a	Involved in the study	
☒	☐	ChIP-seq
☒	☐	Flow cytometry
☒	☐	MRI-based neuroimaging

Antibodies

Antibodies used	Chicken anti-GFP (ab13970, 1:100, Abcam), goat anti-HRP (123-605-021, 1:200, Jackson ImmunoResearch), rabbit anti-RFP (600-401-379, 1:500, Rockland Immunochemicals), rabbit anti-Ca-a1D (1:50, John Y. Kuwada), rabbit anti-Ca-a1D II (1:100, this study), rabbit anti-Ca-b (1:100, this study), rabbit anti-Irk1 (1:100, this study), and fluorescence-conjugated secondary antibodies (1:1000, Jackson ImmunoResearch).
Validation	Antibodies meet all of the quality control standards defined by manufactures. Validation statements for the commercial antibodies are available on the manufactures websites.<br>Chicken anti-GFP (ab13970, Abcam) https://www.abcam.com/products/primary-antibodies/gfp-antibody-ab13970.html<br>goat anti-HRP (123-605-021, Jackson ImmunoResearch) https://www.jacksonimmuno.com/catalog/products/123-605-021<br>rabbit anti-RFP (600-401-379, Rockland Immunochemicals) https://www.rockland.com/categories/primary-antibodies/rfp-antibody-pre-adsorbed-600-401-379/<br>rabbit anti-Ca-a1D (1:50, John Y. Kuwada) Hsu et al., 2020 PNAS<br>The homemade antibodies were confirmed by RNAi knockdown.<br>rabbit anti-Ca-a1D II (1:100, this study) Supplementary Figure 3c, d, g and h<br>rabbit anti-Ca-b (1:100, this study) Supplementary Figure 3e, f, i and j<br>rabbit anti-Irk1 (1:100, this study) Supplementary Figure 6e and f

Animals and other organisms

Policy information about studies involving animals; ARRIVE guidelines recommended for reporting animal research

Laboratory animals	Drosophila melanogaster is used in the study. Both male and female flies at the third instar larval stage were used.
Wild animals	No wild animals were used in the study.
Field-collected samples	No field collected samples were used in the study.
Ethics oversight	All studies and procedures involving animal subjects were performed under the approval of the Institutional Biosafety Committee

(IBC).

Note that full information on the approval of the study protocol must also be provided in the manuscript.
