# Supplementary material for: Glia instruct axon regeneration via a ternary modulation of neuronal calcium channels in Drosophila
Source: Nat Commun. 2023 Oct 14;14:6490. doi: 10.1038/s41467-023-42306-2 (PMC10576831; doi:10.1038/s41467-023-42306-2)
Supplement: Supplementary file 4 — Description of Additional Supplementary Files [file 41467_2023_42306_MOESM4_ESM.docx]

**Description of Additional Supplementary Files**

**Supplementary Movie 1. C4da neurons show axotomy-induced Ca^2+^ transients.** The time lapse was captured at around 4 seconds per frame and the video is rendered at 5 frames per second (fps).

**Supplementary Movie 2. C3da neurons do not show axotomy-induced Ca^2+^ transients.** The time lapse was captured at around 3 seconds per frame and the video is rendered at 5 frames per second (fps).

**Supplementary Movie 3. C3da neurons overexpressing Ca-α1D and Ca-β show axotomy-induced subthreshold transients (STT).** The time lapse was captured at around 3 seconds per frame and the video is rendered at 5 frames per second (fps).
